# Effects of Weather on the Severity of Menstrual Symptoms Among College- and High School-Going Women in the Southern Regions of Saudi Arabia

**DOI:** 10.7759/cureus.66786

**Published:** 2024-08-13

**Authors:** Saeed Abdullah Saeed Alqahtani, Hatim Tagalsir Osman Ali, Faris A Alasmre, Rawabi Fahad Alghamdi, Hind A Alasmre, Lujain A Alasmre, Farah ALMuqrin, Ali A Almuntashiri, Muhannad A Alshahrani, Maryam M Majrashi

**Affiliations:** 1 Obstetrics and Gynecology, Abha Maternity and Children's Hospital, Abha, SAU; 2 Obstetrics and Gynecology, College of Medicine, King Khalid University, Abha, SAU; 3 Internal Medicine, College of Medicine, King Khalid University, Abha, SAU; 4 Obstetrics and Gynecology, King Fahad Hospital, Albaha, SAU; 5 Medicine, College of Medicine, King Khalid University, Abha, SAU; 6 Medicine, Imam Muhammed Ibn Saud Islamic University, Riyadh, SAU; 7 General Practice, Um Sarar Primary Health Care Center, Khamis Mushait, SAU; 8 General Practice, Aseer Central Hospital, Abha, SAU; 9 Medicine, Jazan University, Abha, SAU

**Keywords:** premenstrual syndrome, women health, hot weather, cold weather, fatigue, pain, weather, pms

## Abstract

Background: The effects of weather on the severity of menstrual symptoms have been a topic of interest and research for many years. While some studies have shown a correlation between weather conditions and increased severity of menstrual symptoms, others have found no significant relationship.

Objective: The current study aimed to assess the effects of weather on the severity of menstrual symptoms among women going to college and high school in the southern regions of Saudi Arabia.

Methods: A descriptive cross-sectional study was conducted targeting all women going to college and high school in southern regions of Saudi Arabia. An online questionnaire was used for data collection. The data were entered and analyzed in IBM SPSS Statistics for Windows, version 26 (IBM Corp., Armonk, NY), and stored with no attempts to identify the participants.

Results: The study included 484 participants; most of them were 20-30 years old; 64.5% had regular menstrual cycles. It was observed that mood fluctuations, bloating, difficulty concentrating, breast pain, irritability, anxiety, social isolation, feeling tired, headaches, and mood changes are all affected to some degree before and during menstruation. On the other hand, the amount of blood, duration of the course, physical activity during menstruation, nutritional habits, lower back pain, and sleep disorders showed varying percentages of impact. These findings provide valuable insights into the physiological and psychological changes that occur during the menstrual cycle.

Conclusion: In conclusion, the relationship between weather and the severity of menstrual symptoms is a complex and multifaceted topic. While some women may perceive a correlation between certain weather conditions and an increase in symptom severity, the scientific evidence in support of this connection is still limited and inconclusive. Further research is needed to better understand the underlying mechanisms and to provide evidence-based recommendations for managing menstrual symptoms in relation to weather conditions.

## Introduction

Many years ago, body temperature was described to change with the menstrual cycle by a biphasic rhythm, with body temperature higher in the luteal phase after ovulation [1]. This feature was used by women to track ovulatory cycles and menstrual cycle phases through daily measurement of their body temperature, and they continue to do so with novel temperature sensors that are now available to continuously track 24-hour body temperature rhythms [2]. 

According to previous studies, women's menstrual cycles are often influenced by the weather, and cold temperatures frequently exacerbate the situation for many women [3]. Cold weather probably affects the menstrual cycle, besides other factors including changes in diet, exercise, and sleeping patterns that make premenstrual syndrome (PMS) symptoms more severe [4]. During the colder seasons, many women tend to adopt a more sedentary lifestyle, which can significantly impact their experience of PMS. Modifying diet, increasing physical exercise routines, and using over-the-counter medication can be useful in combating these PMS symptoms [3, 4]. Extremely hot weather wreaks havoc on our cycles, with many of the more frustrating symptoms associated with menstruation being exacerbated by the heat [5]. Literature concluded that while women may not have heavier periods in a heatwave, they may feel worse because hot weather can affect stress levels. The psychological symptoms classically attributed to periods such as low mood, anxiety, and tearfulness rear their ugly heads far quicker than they would on a warm day [6]. The current study aimed to investigate the effects of weather on the severity of menstrual symptoms among college- and high school-going females in southern regions of Saudi Arabia.

## Materials and methods

A descriptive cross-sectional study was conducted targeting all college- and high school-going females in southern regions of Saudi Arabia. The study included all female students attending college and secondary school in the Aseer region during the study period. The study questionnaire covered the participants' sociodemographic data, qualifications, residence area (urban vs. rural), menstrual history, weather at the residence area, changes in menstruation symptoms relative to seasonality (quarterly at a year), and their coping methods. The final questionnaire, after being validated, was uploaded using social media platforms and the internet until no more responses were achieved.

The study's inclusion criteria specify females attending high school or college in the Aseer region who are menstruating and have provided consent for participation. Conversely, exclusion criteria apply to non-menstruating females, individuals living outside the Aseer region, females with diagnosed chronic menstrual disorders, and those who have declined participation in the study. Specifically, medical conditions like thyroid disorders, polycystic ovary syndrome (PCOS), and vitamin D deficiency were excluded from the study to ensure a consistent sample focusing on PMS symptoms in menstruating females without these specific medical conditions.

Data management and analysis

The data were entered and analyzed in IBM SPSS Statistics for Windows, version 26 (IBM Corp., Armonk, NY), and stored with no attempts to identify the subjects because the questionnaire does not include any personal information such as the name, ID number, or any kind of specific personal information that can specify the participant. Qualitative variables are presented as percentages and numbers (mean, frequency, etc.) and shown in the figures.

## Results

Table 1 shows that the majority were aged between 20 and 30 years, accounting for 71.1% of the total study group. The majority of the population was single, with 71.1% of the population being single. The majority of the population attended medical college (53.7%) and non-medical college (46.3%). The majority of the population lived in urban areas, accounting for 71.1% of the population. The climate was characterized by cold winters and mild summers, with the highest percentage experiencing cold winters and mild summers (24.8%). The largest group of individuals in the population had a monthly family income ranging from 5,000 to 10,000 Saudi riyals (40.5%), followed by those earning more than 10,000 riyals (38.8%) and less than 5,000 riyals (20.7%). Non-smokers made up the majority of the population (95.9%), with a small percentage being ex-smokers (1.7%) and current smokers (2.5%). The majority of the population exercised sometimes (67.8%), followed by those who never exercised.

**Table 1:** Sociodemographic characteristics of participants (n = 484)

Parameter		Number	Percentage
Age (years)	Less than 20	76	15.7
	20 - 30	244	71.1
	31 - 40	36	7.4
	41 - 50	28	5.8
Education level	Medical college	260	53.7
	Non-medical college	224	46.3
Marital status	Married	124	25.6
	Single	244	71.1
	Divorced	16	3.3
Place of residence	Urban	244	71.1
	Rural	140	28.9
Weather	Cold in the winter and hot in the summer	40	8.3
	Cold in the winter and mild in the summer	120	24.8
	Cold most of the year	128	26.4
	Hot most of the year	112	23.1
	Mild in the winter and hot in the summer	84	17.4
Family income per month	Less than 5,000 Saudi riyals	100	20.7
	5,000 - 10,000 Saudi riyals	196	40.5
	More than 10,000 thousand Saudi riyals	192	38.8
Smoking	I am not a smoker	464	95.9
	Ex-smoker	8	1.7
	Smoker	12	2.5
Exercise	Never	128	26.4
	Sometimes	328	67.8
	Always	28	5.8
Diet	Health system	40	8.3
	Moderate regime	372	76.9
	Fast food	72	14.9
Age at onset of menstruation (years)	9 - 11	88	18.2
	12 - 15	380	78.5
	16 - 20	16	3.3

Based on the data provided in Table 2, it is evident that the majority of respondents reported having a regular cycle, with 64.5% answering "yes." A smaller percentage, 19.8%, indicated that their cycle was not regular, while 15.7% were unsure. When asked about the duration of their cycle, the majority, 81.8%, reported that it lasts from two to seven days. A smaller percentage, 13.2%, mentioned that their cycle lasted more than seven days, while only 5.0% reported a cycle duration of less than two days. In terms of the amount of bleeding, the majority of respondents, 65.3%, described their bleeding as natural; 18.2% reported heavy bleeding, while 16.5% mentioned irregular bleeding.

**Table 2:** Menstruation regularity and associates among participants (n = 484)

Parameter		Number	Percentage
Regular cycle	Yes	312	64.5
	No	96	19.8
	Maybe	76	15.7
Number of menstruating days	Less than two days	24	5.0
	From 2 to 7 days	396	81.8
	More than 7 days	64	13.2
Amount of bleeding	Heavy	88	18.2
	Natural	316	65.3
	Irregular	80	16.5
Anxiety and stress during menstruation	Intense	132	27.3
	Slight	224	46.3
	None	128	26.4
Use contraceptives	Yes	56	11.6
	No	428	88.4
If the answer is yes, mention the method used	Contraceptive chip	12	25.0
	Pills	24	50.0
	Spiral	20	41.7

Regarding anxiety and stress during menstruation, 46.3% of respondents reported experiencing slight levels, while 27.3% mentioned heavy anxiety and stress. A smaller percentage, 26.4%, stated that they experienced no or normal levels of anxiety and stress. When asked about the use of contraceptives, the majority, 88.4%, reported not using any. However, 11.6% mentioned using contraceptives. Among those who answered "yes," the most commonly used methods were contraceptive pills (COP) (50.0%), followed by the contraceptive chip (25.0%), and the spiral (41.7%).

The data provided in Table 3 show the percentage of individuals who experience various symptoms before and during menstruation, as well as their response to cold weather. Mood fluctuations before menstruation were reported by 74 individuals, which accounts for 30.6% of the total. In contrast, a decrease in mood was observed in 20 individuals (8.3%), whereas 148 individuals (61.2%) did not notice any change in their mood when exposed to cold weather. Similarly, bloating before menstruation was reported by 82 individuals (33.9%), while 18 individuals (7.4%) experienced a decrease in bloating. The majority, 142 individuals (58.7%), reported no change in bloating during cold weather. Difficulty concentrating before menstruation was reported by 58 individuals (24.0%), while 16 individuals (6.6%) experienced a decrease in concentration. The majority, 168 individuals (69.4%), reported no change in their ability to concentrate during cold weather.

**Table 3:** Participants' experience of various symptoms before and during menstruation, as well as their response to cold weather (n = 484)

	It increases with cold weather, n (%)	It decreases with cold weather, n (%)	As usual or not affected by cold weather, n (%)
Mood fluctuations before menstruation	148 (30.6%)	40 (8.3%)	296 (61.2%)
Mood fluctuations before menstruation	112 (23.1%)	84 (17.4%)	288 (59.5%)
Bloating before menstruation	164 (33.9%)	36 (7.4%)	284 (58.7%)
Bloating before menstruation	72 (14.9%)	80 (16.5%)	332 (68.6%)
Difficulty concentrating before menstruation	116 (24.0%)	32 (6.6%)	336 (69.4%)
Difficulty concentrating before menstruation	72 (14.9%)	68 (14.0%)	344 (71.1%)
Breast pain before menstruation	132 (27.3%)	20 (4.1%)	332 (68.6%)
Breast pain before menstruation	60 (12.4%)	68 (14.0%)	356 (73.6%)
Irritability before menstruation	120 (24.8%)	36 (7.4%)	328 (67.8%)
Irritability before menstruation	76 (15.7%)	60 (12.4%)	348 (71.9%)
Anxiety before menstruation	120 (24.8%)	28 (5.8%)	336 (69.4%)
Anxiety before menstruation	68 (14.0%)	60 (12.4%)	356 (73.6%)
Social isolation before menstruation	108 (22.3%)	32 (6.6%)	344 (71.1%)
Feeling tired before menstruation	172 (35.5%)	32 (6.6%)	280 (57.9%)
Headache before menstruation	116 (24.0%)	32 (6.6%)	336 (69.4%)
Menstrual pain	240 (49.6%)	24 (5.0%)	220 (45.5%)
The amount of blood	64 (13.2%)	92 (19.0%)	328 (67.8%)
Duration of the course	48 (9.9%)	44 (9.1%)	392 (81.0%)
Physical activity during menstruation	48 (9.9%)	68 (14.0%)	368 (76.0%)
Mood changes during menstruation	100 (20.7%)	36 (7.4%)	348 (71.9%)
Nutritional habits (food intake/number of meals) during menstruation	136 (28.1%)	56 (11.6%)	292 (60.3%)
Lower back pain	196 (40.5%)	16 (3.3%)	272 (56.2%)
Sleep disorders	128 (26.4%)	40 (8.3%)	317 (65.3%)

Breast pain before menstruation was reported by 66 individuals (27.3%), while 10 individuals (4.1%) experienced a decrease in breast pain. The majority, 166 individuals (68.6%), reported no change in breast pain during cold weather. Irritability before menstruation was reported by 60 individuals (24.8%), while 18 individuals (7.4%) experienced a decrease in irritability. The majority, 164 individuals (67.8%), reported no change in irritability during cold weather.

Anxiety before menstruation was reported by 60 individuals (24.8%), while 14 individuals (5.8%) experienced a decrease in anxiety. The majority, 168 individuals (69.4%), reported no change in anxiety during cold weather. Social isolation before menstruation was reported by 54 individuals (22.3%), while 16 individuals (6.6%) experienced a decrease in social isolation. The majority, 172 individuals (71.1%), reported no change in social isolation during cold weather. Other symptoms such as feeling tired, headaches, lower back pain, and sleep disorders were also reported by a significant number of individuals. 

## Discussion

The effects of weather on the severity of menstrual symptoms have been a subject of interest and research for many years. Menstruation is a natural physiological process that occurs in women of reproductive age, and it is often accompanied by a range of symptoms, including pain, bloating, mood swings, and fatigue. While the exact cause of these symptoms is still not fully understood, it is believed that hormonal fluctuations play a significant role [7].

Weather conditions, such as temperature, humidity, and barometric pressure, have been suggested as potential factors that can influence the severity of menstrual symptoms. Some studies have reported an association between certain weather patterns and an increase in the intensity of symptoms, while others have found no significant relationship. It is important to note that the research in this area is still limited and inconclusive, and further investigations are needed to establish a clear understanding of the relationship between weather and menstrual symptoms [8].

One possible explanation for the perceived impact of weather on menstrual symptoms is the influence of barometric pressure. Barometric pressure refers to the weight of the atmosphere pressing down on the earth's surface. Changes in barometric pressure can affect the body's internal pressure, potentially leading to discomfort and pain. Some women have reported increased menstrual pain and cramping during periods of low barometric pressure, such as before a storm or on rainy days [8, 9]. However, more research is needed to confirm these anecdotal reports and understand the underlying mechanisms.

While previous studies have explored the effects of weather on the severity of menstrual symptoms, the results remain inconclusive. Some studies suggest that higher temperatures, humidity, low barometric pressure, and seasonal changes may contribute to the exacerbation of symptoms. However, the small sample sizes and lack of consistent findings highlight the need for further research in this area. Understanding the relationship between weather and menstrual symptoms could provide valuable insights into the management and treatment of PMS, ultimately improving the quality of life for women worldwide [10].

Our research findings indicate that various symptoms experienced before and during menstruation are influenced by cold weather. Mood swings, bloating, difficulties with concentration, breast tenderness, irritability, anxiety, social withdrawal, fatigue, headaches, menstrual cramps, and sleep disturbances all show a decrease in prevalence during colder temperatures. Conversely, factors such as blood loss, physical activity levels, mood variations, dietary habits, and lower back pain do not appear to be impacted by cold weather conditions. This correlation was supported by a study that revealed that higher temperatures were linked to heightened severity of PMS symptoms [11]. Additionally, another study noted that women reported more intense symptoms during winter months compared to other seasons, with researchers attributing this to reduced sunlight exposure and lower vitamin D levels in the winter season [12]. Furthermore, a separate study found a notable connection between changes in barometric pressure and the severity of menstrual pain, suggesting that a decrease in barometric pressure was associated with increased pain severity in 3,000 women [13]. Another study, involving 1,000 women, revealed that higher humidity and lower temperatures were correlated with elevated menstrual pain severity [14]. Finally, a research study published in the Journal of Obstetrics and Gynaecology Research in 2019, which examined 200 women, highlighted that higher atmospheric pressure was linked to increased severity of menstrual symptoms, specifically pain, fatigue, and mood fluctuations [15]. Previous research has delved into the correlation between weather patterns and symptoms of PMS in women. One study involving 500 women showed that higher temperatures and lower atmospheric pressure were linked to greater severity of PMS symptoms [16]. Similarly, another study examining 300 women reported that higher temperatures and reduced barometric pressure were associated with increased severity of menstrual symptoms, including pain, bloating, and mood changes [8]. A study featured in the British Journal of Obstetrics and Gynaecology in 1998, which included 50 women, discovered that temperature and atmospheric pressure fluctuations were connected to heightened PMS symptoms like irritability, mood swings, and breast tenderness [17]. Additionally, a study focusing on humidity's impact on PMS symptoms revealed that elevated humidity levels were correlated with more severe symptoms, as high humidity could potentially disrupt the body's temperature regulation and exacerbate hormonal imbalances [18]. Furthermore, a study involving 3,000 women found that increased humidity levels were linked to heightened menstrual symptom severity, encompassing pain, bloating, and headaches [19].

In addition to weather conditions, other environmental factors, such as air pollution and allergens, may also contribute to the severity of menstrual symptoms. Exposure to pollutants and allergens can trigger inflammation and exacerbate existing symptoms. Furthermore, stress and changes in routine associated with weather fluctuations, such as disruptions in sleep patterns or alterations in physical activity levels, may also contribute to the perceived impact of weather on menstrual symptoms [8].

While the effects of weather on the severity of menstrual symptoms are still not fully understood, it is crucial to approach this topic with caution and acknowledge the limitations of the available research. It is essential to consider multiple factors, such as hormonal fluctuations, lifestyle, and overall health when assessing the impact of weather on menstrual symptoms.

The study has several potential limitations. These include a small sample size, which may restrict the generalizability of the findings to a broader population. Sampling bias could arise if the participants are not fully representative of all college- and high school-going females in the southern regions of Saudi Arabia. Additionally, relying on self-reported menstrual symptoms may introduce biases or inaccuracies in the data. The cross-sectional design of the study may hinder establishing causality between weather conditions and menstrual symptoms, as it does not consider changes over time. Furthermore, the study may not adequately address all potential confounding variables that could influence the severity of menstrual symptoms, such as lifestyle factors or underlying health conditions. The findings' generalizability may be limited due to the study's specific focus on college- and high school-going females in southern regions, of Saudi Arabia. Cultural factors unique to Saudi Arabia could influence how menstrual symptoms are experienced and reported, potentially affecting the study results. Issues related to participant compliance, such as incomplete data or dropout rates, could also impact the validity of the findings. Moreover, there is a risk of publication bias, where studies with negative or inconclusive results are less likely to be published, potentially affecting the overall understanding of the relationship between weather and menstrual symptoms.

## Conclusions

In conclusion, the majority of respondents had a regular menstrual cycle, with most describing it as natural. They experienced anxiety and stress during menstruation, with contraceptives being the most common method. Symptoms like mood fluctuations, bloating, concentration, breast pain, irritability, anxiety, and social isolation were reported by the majority of participants.

The relationship between weather and the severity of menstrual symptoms is a complex and multifaceted topic. While some women may perceive a correlation between certain weather conditions and an increase in symptom severity, the scientific evidence in support of this connection is still limited and inconclusive. Further research is needed to better understand the underlying mechanisms and to provide evidence-based recommendations for managing menstrual symptoms concerning weather conditions. In the meantime, women must listen to their bodies, seek appropriate medical advice, and implement individualized strategies for managing their menstrual symptoms, regardless of weather conditions.
